# Effect of Boron Content in LiOH Solutions on the Corrosion Behavior of Zr-Sn-Nb Alloy

**DOI:** 10.3390/ma17102373

**Published:** 2024-05-15

**Authors:** Yongfu Zhao, Zongpei Wu, Zirui Chen, Zhaohui Yin, Min Tang, Jing Xiong, Ping Deng

**Affiliations:** Nuclear Power Institute of China, Chengdu 610213, China; pepenpic@aliyun.com (Z.W.); raymix@aliyun.com (Z.C.); 136509242@163.com (Z.Y.); tang.m@163.com (M.T.); 15852492869@163.com (J.X.); dengping13s@126.com (P.D.)

**Keywords:** boron content, LiOH solutions, corrosion, Zr-Sn-Nb alloys

## Abstract

In pressurized water reactors, LiOH may be concentrated in some areas, leading to the accelerated corrosion of fuel claddings. Injecting boric acid into primary coolants can mitigate the accelerated corrosion effect of LiOH on Zircaloys, but the effects of boron content on the corrosion behavior of the Zr-Sn-Nb alloy are still unknown. This work focused on the corrosion and hydrogen absorption behavior at 360 °C/18.6 MPa in 100 mg/kg LiOH solutions with 0 mg/kg, 50 mg/kg, and 200 mg/kg boron contents for up to 510 days, aiming to study the effect of boron content on corrosion resistance in LiOH solutions. Corrosion kinetics, microstructures of oxide films, hydrogen absorption concentrations and hydride morphology were obtained after the test. The results show that injecting boron in LiOH solutions can significantly reduce the corrosion weight gain, hydrogen concentration, and hydrogen length of Zr-Sn-Nb alloys, that is, improving corrosion resistance effectively. During the oxidation of the Zr-Sn-Nb alloy, B^3+^ and Li^+^ incorporate in oxide films. The incorporation of Li^+^ may lead to the generation of oxygen vacancies, which can carry oxygen from the solutions to O/M interface, accelerating corrosion. The incorporation of B^3+^ in oxide films will slow down the oxidation of Zr-Sn-Nb alloys by reducing the oxygen vacancies caused by Li^+^ aggregation.

## 1. Introduction

Zirconium (Zr) alloys, also well known as Zircaloys, are currently the only cladding material used in water-cooled reactors due to its low thermal neutron capture cross-section, excellent radiation resistance, reasonable mechanical properties and unique corrosion resistance in high temperature water [1,2,3]. Traditional Zircaloys alloys, such as the Zr-2 and Zr-4 alloy, were widely used in water-cooled reactors from the 1950s to the 1990s [3]. Zr-2 alloy with nominal composition Zr-1.5wt%Sn-0.15wt%Fe-0.1wt%Cr-0.05wt%Ni showed good corrosion resistance, and had been applied to boiling water reactors (BWRs) and pressurized water reactors (PWRs) as fuel claddings. However, Ni in the Zr-2 alloy led to hydrogen embrittlement. To solve this problem, Zr-4 alloy with nominal composition Zr-1.5wt%Sn-0.2wt%Fe-0.1wt%Cr was developed. Zr-Nb alloys, such as the M5 alloy, had excellent corrosion and hydrogen absorption resistance [4], are still the first choice for the cladding materials of many PWRs. With the development of PWRs toward deepening the burn-up of nuclear fuel and extending the reload cycle, higher requirements are put forward for the performance of zirconium alloys. Thereupon, Zr-Sn-Nb alloys with better corrosion, radiation, creep, and fatigue resistance have been developed [5].

At present, most of the primary coolant of PWRs is boron–lithium type, and the irradiation decomposition of water is suppressed by dissolved hydrogen [6]. Under this condition, Zr-Sn-Nb alloy maintains good corrosion resistance. However, LiOH may be concentrated in some areas, and has a negative effect on the corrosion resistance of zirconium alloys. The effect of Li^+^ concentrations (within the range of 2.2–7000 mg/kg) on the corrosion resistance of Zr-4 has been studied [7,8], and the following phenomena were found. Firstly, when the Li^+^ concentration was only a few mg/kg, the corrosion behavior of Zr-4 alloy is almost the same as that in pure water. Secondly, when the Li^+^ concentration was tens of mg/kg, the corrosion transition was earlier, and the corrosion after transition was accelerated. Thirdly, when the Li^+^ concentration increased to 350 mg/kg, the stage before corrosion transition was no longer measurable, and the corrosion rate continued to increase after corrosion transition. Injecting boric acid could mitigate the accelerated corrosion effect of LiOH on Ziraloys [9]. Although autoclave corrosion tests have shown that the corrosion resistance of the Zr-Sn-Nb alloy in LiOH solutions was better than Zircaloys [10], there was still a lack of research on the effect of injecting boron on the Zr-Sn-Nb alloy.

There are three understandings of the deterioration of corrosion resistance induced by Li^+^. Firstly, Li^+^ replaces the position of Zr^4+^ in ZrO_2_ lattice, resulting in the generation of additional oxygen vacancies and promoting oxygen diffusion [11]. Secondly, Li^+^ promotes the recrystallization of ZrO_2_, leading to an increase in grain boundary and the formation of pores and cracks in oxides [12]. Thirdly, Li^+^ increases the porosity of oxide films, leading to the preferential dissolution of connection positions in the ZrO_2_ lattice, causing the penetration of protective oxide films, ultimately leading to the rapid diffusion of the oxygen-to-oxide/metal (O/M) interface [13]. Xie et al. [14] found the aggregation of Li at the ZrO_2_ grain boundary, and they believed it reduced the grain boundary energy of oxide film, resulting in defects and cracking.

As for the corrosion inhibition mechanism of injecting boron, there are also three understandings. Firstly, the reaction between boric acid and the -OLi group on the surface may alleviate the corrosion problem caused by Li^+^ concentration [15]. Secondly, boron promotes the formation of oxides from dissolved alloy elements, covering the pores in ZrO_2_ and hindering corrosion [16]. Thirdly, Boron changes the chemical environment in the pores of ZrO_2_, reducing the solubility of oxides [17]. However, there is still a lack of clear conclusions about the corrosion inhibition mechanism of injecting boron.

To sum up, LiOH is a commonly used non-volatile alkaline agent in PWRs. It poses a risk of local concentration on the surface of fuel cladding, which can lead to the accelerated corrosion of zirconium alloy. Therefore, it is considered to inject a certain concentration of boric acid to mitigate the effects of Li^+^. However, the mechanism by which zirconium alloy accelerated the corrosion caused by the local concentration of LiOH is still unclear, and the laws of boric acid alleviating Zr-Sn-Nb alloy corrosion are also unknown. In order to clarify the influence of injecting boron on the corrosion resistance of Zr-Sn-Nb alloy, this paper studied the corrosion behavior of the Zr-Sn-Nb alloy in concentrated LiOH solutions with different boron concentrations, and an attempt has been made to explain the corrosion inhibition mechanism of zirconium alloys by injecting boron to the aqueous solutions of the primary loop of PWRs.

## 2. Materials and Methods

### 2.1. Alloy and Materials

In this study, we selected a Zr-Sn-Nb alloy plate with a thickness of (2.1 ± 0.1) mm as a material. We obtain Zr-Sn-Nb plate from State Nuclear Bao Ti Zirconium Industry Co., Ltd., Baoji, China and the composition is shown in Table 1.

### 2.2. Corrosion Experiment

The corrosion experiment was conducted using an autoclave. Plates were cut into (30 ± 1) mm × (20 ± 1) mm × (1.4 ± 0.1) mm sheets. Before corrosion, specimens were pickled in the standard metallographic etching solution for about 1 min to obtain a shiny surface. These specimens were moved immediately into the flowing water after pickling. Rinsing in the flowing water lasted for about 10 min and then specimens were transferred into the boiling deionized water for another 10 min to eliminate the influence of fluorine. The corrosion test was carried out in a static autoclave at 360 ± 3 °C/18.6 ± 1.4 MPa following the ASTM-G2/G2M-06 standard [18]. The autoclave was deaerated each time during heating-up at 140–150 °C. After deaeration, the autoclave was heated to 360 ± 3 °C, and then the corrosion test started. The aqueous solution compared in this study is shown in Table 2. By injecting 50 mg/kg and 200 mg/kg of boron to the concentrated LiOH solutions, the effects of different boron concentrations on the corrosion resistance of the Zr-Sn-Nb alloy were compared. Specimens were taken and weighed every 30 days, and corrosion resistance was evaluated by weight gain. In this work, at least three parallel samples were left in the autoclave to ensure an average measuring of weight gain.

### 2.3. Microscopic Analysis after Corrosion

Scanning electron microscope (SEM) was used to observe the surface and cross-sectional morphology of oxide films. Transmission electron microscope (TEM) was used to obtain a more detailed local microstructure of the oxide films. The thin foils of cross-sections of the oxide films were prepared using focused ion beam (FIB). X-ray diffraction (XRD) was used to analyze the crystal structure of the sample after corrosion testing. Atom probe tomography (APT) was used to analyze the aggregation of elements in the microzone of the oxide film. Glow discharge optical emission spectrometry (GDOES) was conducted to measure the distributions of elements in oxide films along the depth direction. The oxygen/nitrogen/hydrogen determinator was used to analyze the hydrogen contents of the corroded specimens, and the hydrides were observed in the matrix of the corroded specimens using an optical microscope.

## 3. Results

### 3.1. Effect of Boron Injection on Corrosion Behavior of Zr-Sn-Nb Alloy

#### 3.1.1. Corrosion Kinetics

**Figure 1:**
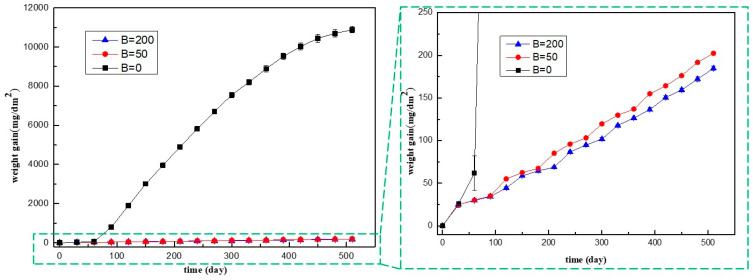
Corrosion kinetics of Zr-Sn-Nb alloy corroded in Li = 100 mg/kg solutions with, 50, 200 mg/kg boron, black square data represent the weight gain of samples corroded in B = 0 mg/kg solutions, red circular data represent the weight gain of samples corroded in B = 50 mg/kg solutions, blue triangle data represent weight gain of samples corroded in B = 200 mg/kg solutions.

The weight gain profile of Zr-Sn-Nb alloy is shown in Figure 1. The injection of boron significantly reduced the corrosion rate of Zr-Sn-Nb alloy in aqueous solutions with a Li concentration of 100 mg/kg. In B = 0 mg/kg solutions, the corrosion transition of Zr-Sn-Nb alloy was occurred in 60 days, after that, the corrosion rate increased sharply, and the weight gain was more than 10,000 mg/dm^2^ in 420 days. In B = 50 mg/kg and B = 200 mg/kg solutions, the corrosion rates were much lower than that in B = 0 mg/kg solutions, and after 510 days, the weight gain was 202.38 mg/dm^2^ and 184.77 mg/dm^2^, respectively, which was only 1/50 of that in B = 0 mg/kg solutions. It is clear that the corrosion rate of Zr-Sn-Nb alloy decreases significantly after increasing the B concentration from 0 to 50 mg/kg. When the B content was increased from 50 mg/kg to 200 mg/kg, the corrosion inhibition effect was significantly reduced. After 510 days, the weight gain of the Zr-Sn-Nb alloy in B = 200 mg/kg solutions was only about 18 mg/dm^2^ lower compared to that in B = 50 mg/kg solution.

#### 3.1.2. Top Appearance

**Figure 2:**
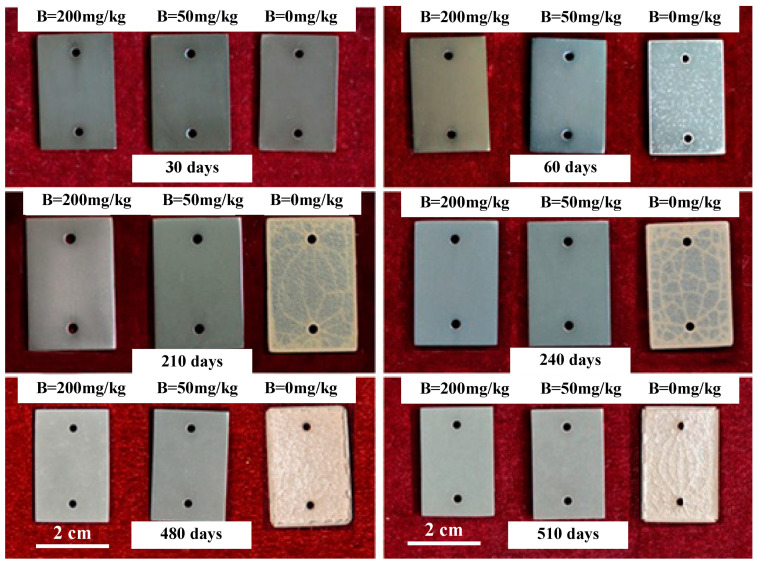
Top appearance of the Zr-Sn-Nb alloy in different solutions at different corrosion times, the solutions samples corroded in are labeled in the upper part of sub-figures, and the corrosion times are labeled in the bottom part of sub-figures.

The photograph of corrosion specimens are shown in Figure 2. In B = 200 mg/kg and B = 50 mg/kg solutions, the surface of Zr-Sn-Nb alloy remained uniformly grey-black throughout the corrosion test, and there were no obvious defects on surface. In B = 0 mg/kg solutions, the specimen only showed uniform grey-black color in the first 30 days. After 60 days, a small number of white spots began to appear, and gradually increased and covered the whole surface with the prolongation of the test time. After 210 days, cracks appeared on the surface of specimen. After 480 days, severe cracking occurred in the oxide films.

#### 3.1.3. SEM Morphology of Oxide Films

**Figure 3:**
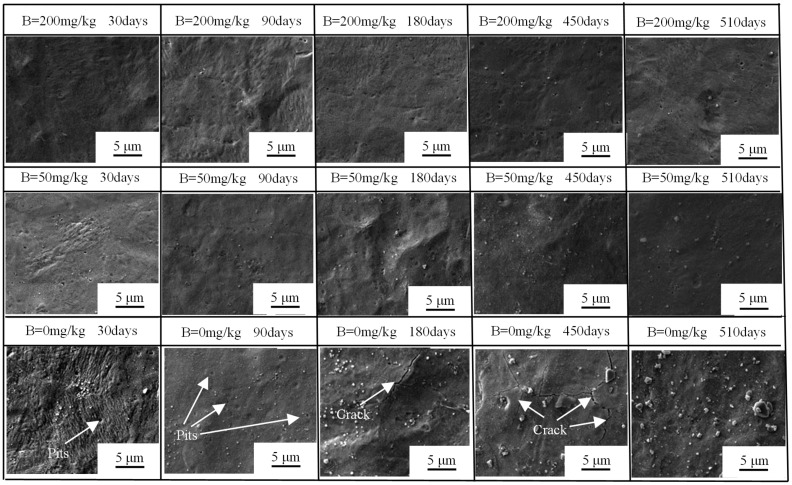
SEM surface morphology of Zr-Sn-Nb alloy in different solutions at different corrosion times, the solutions samples corroded in and corrosion time are labeled in the upper part of sub-figures.

The SEM surface morphology of oxide films are shown in Figure 3. In B = 200 mg/kg and B = 50 mg/kg solutions, the oxide films were uniform and dense. In B = 0 mg/kg solutions, the oxide films present a large number of groove corrosion morphology after 30 days, which may be related to plate textile [19]. After 180 days, cracks began to appear. Comparing the surface morphologies of oxide films in different solutions after the same cycle test, it can be found that there are a large number of oxide particles and a small number of corrosion pits on the surface in B = 0 mg/kg solution.

**Figure 4:**
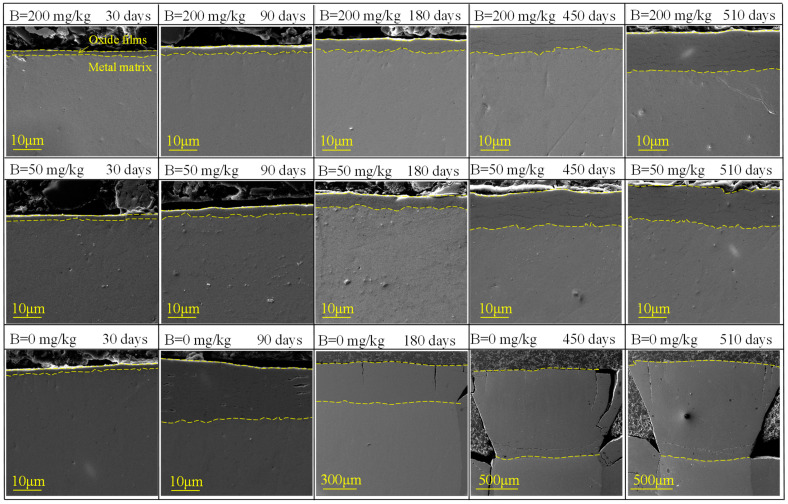
SEM cross-sectional morphology of Zr-Sn-Nb alloy in different solutions at different corrosion times, the solutions samples corroded in and corrosion time are labeled in the upper part of sub-figures.

The SEM cross-sectional morphology of Zr-Sn-Nb alloy corroded for several days (containing Zr matrix and oxide films) are shown in Figure 4. In B = 0 mg/kg solutions, a large number of cracks appeared in oxide films after 90 days. In B = 200 mg/kg and B = 50 mg/kg solutions, cracks appeared in oxide films after 360 and 450 days, respectively. Inhomogeneous oxidation of Zr-Sn-Nb alloys leads to the accumulation of stress in oxide films, which may destroy oxide films and cause cracks [20]. Inhomogeneous oxidation was more severe in B = 0 mg/kg solution, resulting in cracking of oxide films after 90 days corrosion, which was earlier than that in B = 200 mg/kg and B = 50 mg/kg solutions. In addition, after 180 days, cracks perpendicular to surface had appeared in oxide films in B = 0 mg/kg solution, indicating that stress generated by the growth of oxide films led to brittle cracking, and such cracks directly promoted the mass transfer process from surface to O/M interface, accelerating corrosion. As can be seen in Figure 4, with the extension of the test time, the cracks continued to expand to O/M interface, while no cracks appeared until the end of the 510-day test in B = 200 mg/kg and B = 50 mg/kg solutions.

#### 3.1.4. Hydrogen Absorption Concentrations

The hydrogen absorption concentrations of Zr-Sn-Nb alloy in different solutions are shown in Table 3. In B = 0 mg/kg solutions, hydrogen absorption concentrations increased sharply to 400 mg/kg after 90 days, and to 3900 mg/kg after 510 days. In B = 200 mg/kg and B = 50 mg/kg solutions, the hydrogen absorption concentrations was lower than that in B = 0 mg/kg solution. When boron concentration was increased from 50 mg/kg to 200 mg/kg, the hydrogen absorption concentration was slightly decreased.

#### 3.1.5. Hydrides Morphology

**Figure 5:**
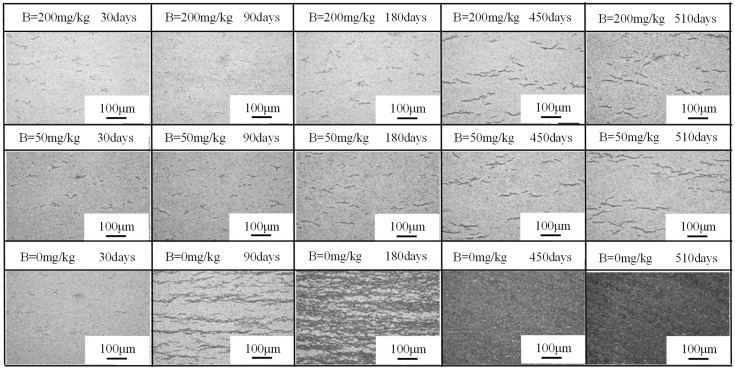
Hydride morphology of Zr-Sn-Nb alloy in different solutions at different corrosion times, the solutions samples corroded in and corrosion time are labeled in the upper part of sub-figures.

After chemical etching, hydrides morphology is shown in Figure 5. At the beginning of the corrosion test, the hydrides length and density in B = 200 mg/kg solutions was lower than that in B = 50 mg/kg and B = 0 mg/kg solutions. As time went on, the hydrides length and density was increased, which tended to be similar in the B = 200 mg/kg and B = 50 mg/kg solutions. In B = 0 mg/kg solutions, hydrides had a tendency to interconnect after 90 days, and then hydrides’ density increased sharply until it covered the whole observation field of view.

### 3.2. Effect of Boron Injection on Microstructure of Oxide Films of Zr-Sn-Nb Alloys

#### 3.2.1. Cross-Sectional Microstructures of Oxide Films

**Figure 6:**
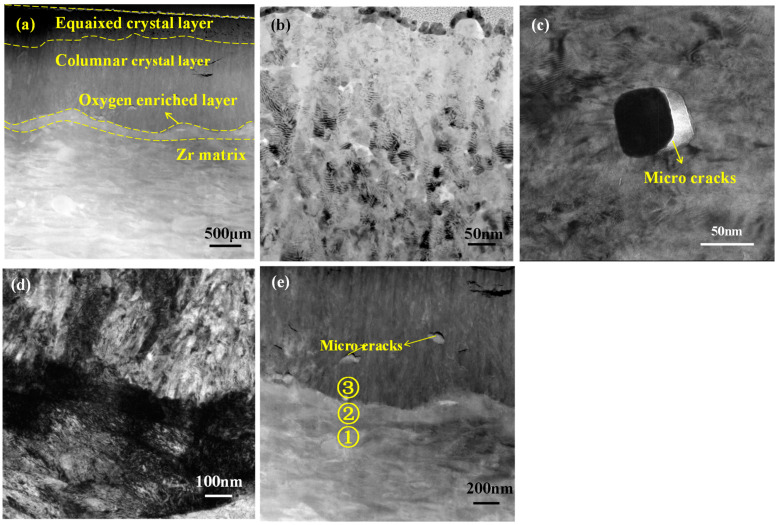
Cross-sectional microstructure of oxide films corroded in B = 200 mg/kg solutions for 30 days: (**a**) cross-section morphology of oxide film; (**b**) pores between equiaxed crystals; (**c**) SPPs observed near O/M interface; (**d**) bright field image of oxygen enriched layer; and (**e**) HAADF image of oxygen-enriched layer.

The oxide films microstructure of Zr-Sn-Nb alloy corroded in B = 200 mg/kg solutions for 30 days (studied by TEM) are shown in Figure 6. From Figure 6a, oxide films were composed of an equiaxed crystal layer, a columnar crystal layer and an oxygen-enriched layer. The outer films were porous, and the thickness was about 200 nm, which consisted of equiaxed crystals with the size of 20–50 nm. The presence of pores between equiaxed crystals indicates that the outer films were not protective, as shown in Figure 6b. Below the equiaxed crystal layer was a columnar crystal layer with a thickness of 1.2 μm. The columnar crystal grew perpendicular to surface with a width of 50–100 nm. To reduce stress accumulation, zirconium alloys preferentially form columnar crystal oxides, and this oxide films can prevent the flow of corrosive media to O/M interface [21]. Near O/M interface, some island-shaped second phase particles (SPPs) with sizes of 50–200 nm could be observed. They are Zr(Nb,Fe,Cr)_2_, and all of them cracked on the side near surface, as shown in Figure 6c. Zr in Zr(Nb,Fe,Cr)_2_ were preferentially oxidized, followed by Fe, Cr, and Nb. The Pilling–Bedworth ratio (PB ratio) of Fe is 2.05, Cr is 2.00, Nb is 2.67. They are larger than that of Zr. So in the subsequent oxidation, the volume expansion of SPPs is inconsistent with that of Zr, generating additional stress and leading to cracking around SPPs [22]. An oxygen-enriched layer with a thickness of 200 nm also existed near O/M interface, and the morphology was shown in Figure 6d,e. Performing electron spectroscopy point scanning at position 1, 2 and 3 in Figure 6e, the results are shown in Table 4. Oxygen-enriched layer can effectively prevent the further diffusion of oxygen into Zr matrix by continuously dissolving oxygen atoms.

**Figure 7:**
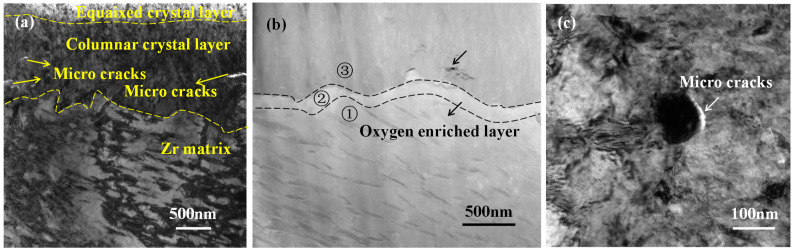
Cross-sectional microstructure of oxide films corroded in B = 0 mg/kg solutions for 30 days: (**a**) cross-section morphology of oxide film; (**b**) morphology of O/M interface; and (**c**) SPPs observed near O/M interface.

Figure 7 shows the cross-sectional microstructure of oxide films corroded in B = 0 mg/kg solutions for 30 days. The oxide films consisted of an equiaxed crystal layer, a columnar crystal layer, and an oxygen enriched layer, which were consistent with the cross-sectional microstructure corroded in B = 200 mg/kg solutions. This indicates that the corrosion mechanism has not changed in different solutions. From Figure 7a, it can be seen that cracks appeared in oxide films. This type of crack was usually caused by stress. When the local stress in oxide films exceeds the limit, cracks parallel to the surface will occur. The local stress was usually caused by uneven oxidation. In B = 0 mg/kg solutions, the Zr-Sn-Nb alloy exhibits significant uneven oxidation leading to oxide cracking, which means that the concentrated Li aggravates the inhomogeneous oxidation of Zr-Sn-Nb alloys. Table 5 shows electron spectroscopy results of positions 1, 2, and 3 in Figure 7b. There is also a oxygen-enriched layer nearby the O/M interface, which is coincided with the result of Figure 6.

#### 3.2.2. Crystal Structure of Oxide Films

**Figure 8:**
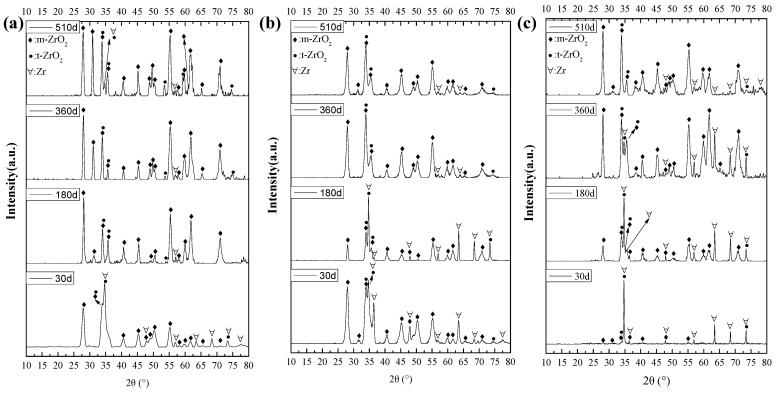
XRD results of Zr-Sn-Nb alloys immersed in different solutions at different times: (**a**) B = 200 mg/kg, (**b**) B = 50 mg/kg, (**c**) B = 0 mg/kg, the corrosion times are labeled in the upper left corner of sub-figures.

XRD was performed to obtain the crystal structure of oxide films, and the results are shown in Figure 8. All the XRD results showed diffraction peaks of Zr [23]. After 30-day corrosion, the oxide films in B = 0 mg/kg solutions was mainly monoclinic ZrO_2_ (m-ZrO_2_) [24], and the oxide films in B = 50 mg/kg solutions were composed of m-ZrO_2_ and tetragonal ZrO_2_ (t-ZrO_2_) [25]. In B = 200 mg/kg solutions, the oxide films were mainly composed of t-ZrO_2_. After being corroded for 180 days and 360 days, the oxide films in B = 0 mg/kg and B = 50 mg/kg solutions mainly consisted of m-ZrO_2_ and contained a small amount of t-ZrO_2_, and oxide films in B = 200 mg/kg solutions were made up of m-ZrO_2_ and t-ZrO_2_. After being corroded for 510 days, the oxide films in B = 0 mg/kg, B = 50 mg/kg, and B = 200 mg/kg solutions were mainly made up of m-ZrO_2_. However, the number of m-ZrO_2_ diffraction peaks in B = 200 mg/kg and B = 50 mg/kg solutions is less than that in B = 0 mg/kg solutions.

During the oxidation of zirconium alloys, t-ZrO_2_ was formed firstly, and then t-ZrO_2_ is converted into m-ZrO_2_ [3]. Therefore, the XRD results show that the oxidation of the Zr-Sn-Nb alloy is effectively inhibited in B = 200 mg/kg solutions. However, in all solutions, the oxidation process of Zr-Sn-Nb did not show significant changes, with the difference being the change in corrosion rate.

#### 3.2.3. Distribution of Elements in Oxide Films

**Figure 9:**
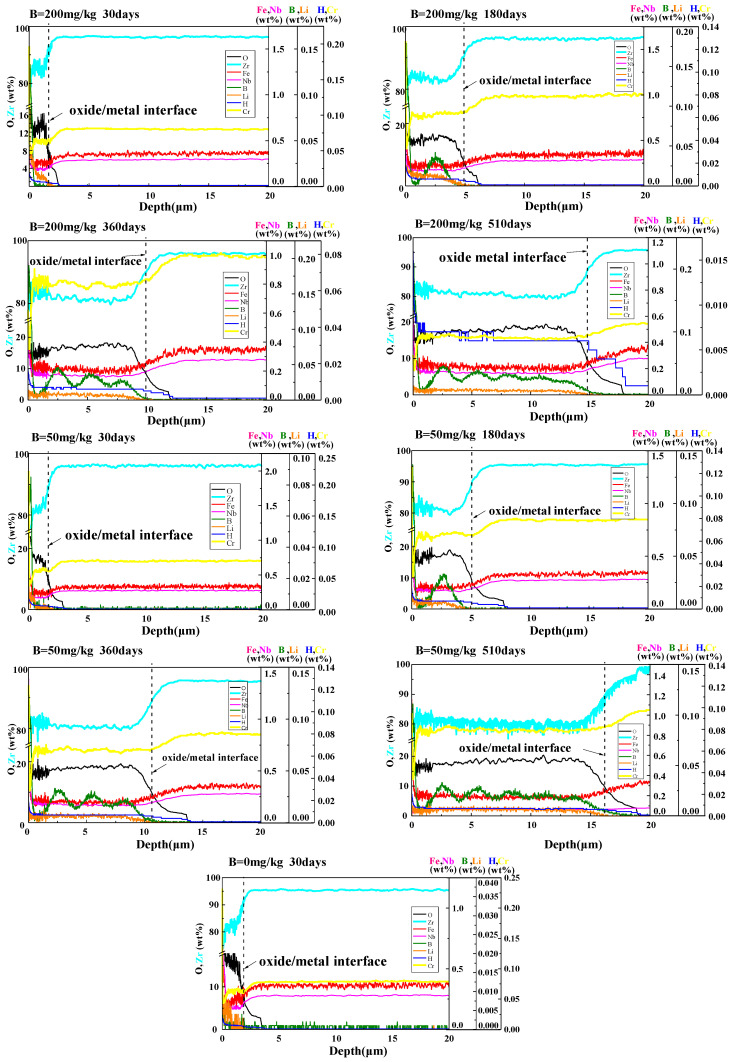
Element distribution along depth direction in oxide films in different solutions at different times, the solutions’ samples corroded in and the corrosion times are labeled in the upper left corner of sub-figures.

GDOES was used to obtain the element distribution along the depth direction in oxide films, and the results are shown in Figure 9. In B = 0 mg/kg solutions, Li infiltrated into oxide films and Li concentration decreased with depth. In B = 50 mg/kg and B = 200 mg/kg solutions, B and Li infiltrated into oxide films, and the infiltration of B did not affect the infiltration of Li. The variation of B concentration showed a periodic wave shape law. The variation pattern of Li concentration is different from B. These indicate that a corrosion transition may have an impact on B distribution in oxide films, while it has no effect on Li distribution.

**Figure 10:**
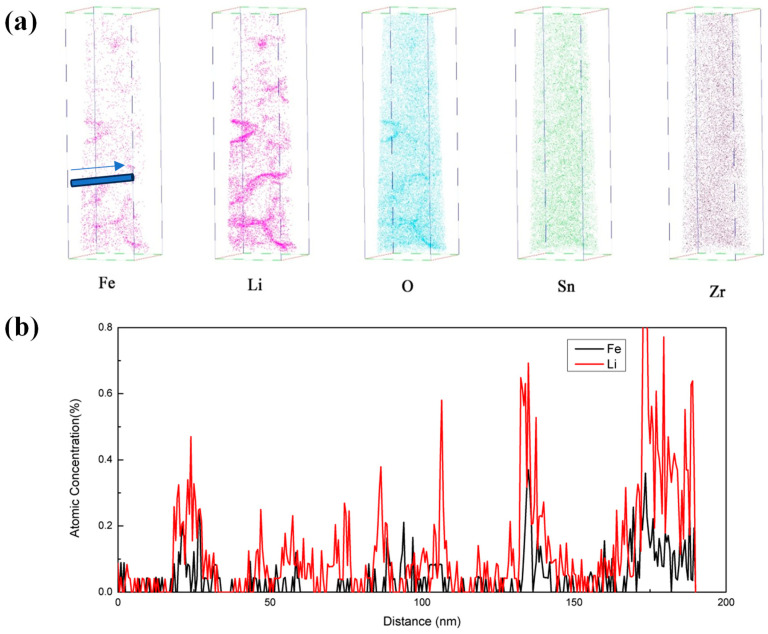
APT results of oxide films corroded in B = 200 mg/kg solutions for 510 days: (**a**) APT three-dimensional reconstruction of the distribution of elements in oxide films, (**b**) distribution of Fe and Li concentrations along the arrow direction in the sub-figure (**a**).

APT was conducted to study the local element segregation in oxide films, and the element distribution of oxide films corroded in B = 200 mg/kg solutions for 510 days are shown in Figure 10. Fe, Li, and O accumulated in a certain area of oxide films, while Zr and Sn were uniformly distributed in the entire oxide. B was not detected, which may be related to the low B concentration. Taking a cylindrical area (with a diameter of 20 nm) along the direction indicated by the arrow in Figure 10a, and the concentration distribution of Fe and Li are shown in Figure 10b. Li and Fe gathered at the same location.

Fe usually exists in the form of Zr(Nb,Fe,Cr)_2_ in Zr-Sn-Nb alloys [2]. This kind of SPPs has good corrosion resistance and will oxidize later than the Zr matrix. Due to the extremely low solid solubility of Fe in ZrO_2_, when Zr matrix around Zr(Nb,Fe,Cr)_2_ is oxidized, Fe will be discharged and aggregated at SPPs boundaries [26]. Meanwhile, others have also found the phenomenon of Fe segregation at oxide grain boundaries [27]. There are different opinions on how Li and O enter oxide films and how they affect the corrosion resistance of zirconium alloys. Research has confirmed that Li in oxides films exists in the form of LiOH • H_2_O or LiOH, and most of Li exists in porous oxides [28]. Xie et al. [14] found that Li will aggregate at grain boundaries during corrosion. Based on the research results mentioned above, the process of Li accelerated corrosion is as follows: Li^+^ and OH^-^ diffuse inward along pores or grain boundaries of oxides, and continuously aggregate at grain boundaries and pores. The size of Li^+^ is 76 pm and the size of Zr^4+^ is 72 pm. When a large amount of Li^+^ aggregate at grain boundary, it is easy to replace the Zr^4+^ in ZrO_2_, leading to an increase in oxygen vacancy. The oxygen vacancies promote the diffusion of H, O, and another corrosion medium along grain boundaries, accelerating corrosion.

In order to analyze the influence of boron on the segregation behavior of Li and Fe, the APT analysis of oxide films corroded in B = 200 mg/kg solutions was compared to that in LiOH = 1 mol/L solutions (no boron injected) conducted by Xie et al. [14]. It was found that the injection of boron did not slow down the segregation of Li and Fe at grain boundaries. In contrast, the peak concentration of Li at grain boundaries (0.6%~0.8%) was higher than that in B = 0 mg/kg solutions (0.1%), which may be related to the longer corrosion time. The peak concentration of Fe at the grain boundary (0.4%) is approximately equal to that in B = 0 mg/kg solutions (0.4%). Based on the above analysis, it can be concluded that the injection of boron will not affect the segregation behavior of Li and Fe.

### 3.3. Accelerated Corrosion Mechanism Induced by Li and Corrosion Inhibition Mechanism of Injecting Boron

The weight gain, the length and concentration of hydrides in B = 50 mg/kg and B = 200 mg/kg solutions was much lower than that in B = 0 mg/kg solutions. These indicate that the injection of boron inhibits the corrosion of Zr-Sn-Nb alloys in concentrated Li solutions. The oxide morphology, composition, and structure of Zr-Sn-Nb alloys in B = 0 mg/kg, B = 50 mg/kg and B = 200 mg/kg solutions showed no significant differences, which means injecting boron did not change corrosion process. It was found that B and Li can infiltrate the oxide films, and Li exhibits an aggregation effect in oxides. This indicates that boron injection does not affect the infiltration and aggregation behavior of Li.

In order to clarify the effects of pH, undissolved LiOH and Li^+^ on corrosion of Zr-Sn-Nb alloy, we calculate pH_360°C_, undissolved LiOH and Li^+^ concentration in different solutions (aqueous solutions in this paper and in other literature) and compare corrosion resistance of zirconium alloys, as shown in Table 6. When the Li concentration exceeds 70 mg/kg, if boron is not injected to the solution, Li will significantly accelerate the corrosion of zirconium alloys. When the B concentration exceeds 1/3 of Li concentration, injecting boron will have a good corrosion inhibition effect. However, increasing B concentration after exceeding the critical concentration cannot enhance the corrosion inhibition effect. In addition, When the Li concentration exceeds 700 mg/kg, injecting boron cannot slow down the accelerated corrosion effect. It also can be found that the Li-induced accelerated corrosion is not related to pH_360°C_, undissolved LiOH, and Li^+^ concentration.

In the three solutions studied in this paper, the Li concentration was basically the same, indicating that the accelerated corrosion is not the sole effect of Li. Although zirconium alloys undergo accelerated corrosion in LiOH solutions, accelerated corrosion does not occur in LiNO_3_, NaOH, KOH, RbOH, and CsOH solutions [7], indicating that single OH^−^ cannot accelerate corrosion. Therefore, the accelerated corrosion in LiOH solution is the result of the cooperative effect of Li^+^ and OH^−^.

According to the Macdonald model [29], the oxide film exhibits semiconductor properties in aqueous solutions. Anionic vacancies in oxides conduct oxygen from aqueous solutions to O/M interface, leading to the oxidation of the matrix, where the concentration of oxygen vacancies determines the rate of oxidation. According to the cross-sectional microstructure, oxide films of Zr-Sn-Nb alloys are consist of equiaxed crystal oxide (outermost), columnar crystal oxide (middle), and oxygen-enriched layer (bottom).

**Figure 11:**
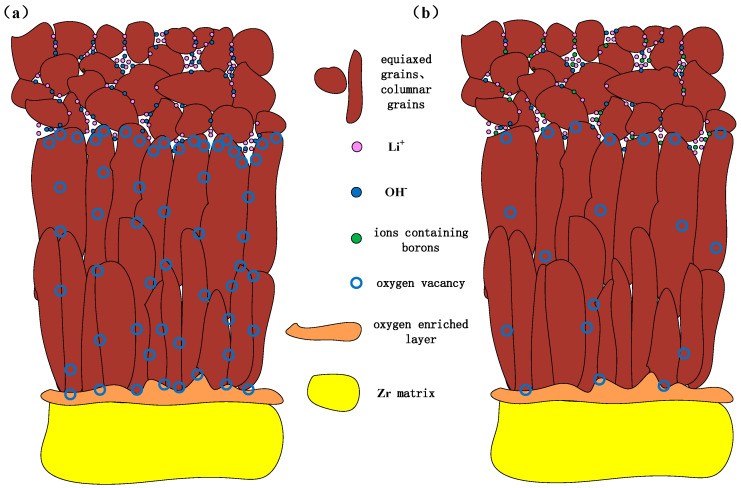
Schematic diagram of mechanism: (**a**) LiOH accelerating corrosion and (**b**) corrosion inhibition of injecting boron.

Based on the discussions mentioned above, we propose the accelerated corrosion mechanism induced by the Li and corrosion inhibition mechanism of injecting boron, as shown in Figure 11. In B = 0 mg/kg solutions, Li^+^ and OH^-^ accelerate corrosion through the six following steps:

1. Li^+^ and OH^−^ diffuse from aqueous solutions to the top of columnar crystal through pores of equiaxed crystal. Moreover, the concentration of corrosive medium is prone to occur in these pores, leading to the enrichment of Li^+^ and OH^−^ at the top of columnar crystals.
2. Li^+^ and OH^−^ diffuse along the grain boundaries of columnar crystal towards by solid-state diffusion, resulting in a uniform distribution of Li in the depth direction. In oxide films, columnar crystals also contain micropores [30]. Therefore, Li^+^ and OH^-^ also tend to be enriched near the micropores.
3. Oxides exhibit acidity or neutrality in solutions, while their surfaces exhibit electronegativity in alkaline solutions [31], requiring the neutralization of cation in solutions. OH^-^ enriched at the top of columnar crystal makes the local environment appear alkaline, promoting negative charge accumulate on the surface of the nearby columnar crystal. At this time, Li^+^ adsorbs on the surface of columnar crystal, which is conducive to its diffusion to the interior of columnar crystal.
4. When Li^+^ is adsorbed on the surface of columnar crystal, it can easily replace Zr^4+^ in ZrO_2_ due to the very close size of Li^+^ and Zr^4+^. In addition, Li^+^ continuously diffuse along grain boundaries to the O/M interface.
5. When Li^+^ replaces Zr^4+^, 1.5 oxygen vacancies form near it [32]. Therefore, due to the presence of a large amount of Li^+^ at the top of columnar crystals, grain boundaries and micropores, a large number of oxygen vacancies will also be generated nearby.
6. Oxygen in solutions can occupy these oxygen vacancies easily through short-range diffusion, resulting in an excess of oxygen in ZrO_2_ lattice. These oxygen diffuse rapidly along grain boundaries to O/M interface through solid-state diffusion, accelerating corrosion [33].

After injecting boron to solutions, the corrosion rate decreased significantly. According to the calculation results in Table 4, the injection of boric acid did not change pH, that is, the alkaline environment did not change, and did not affect the Li^+^ concentration in solutions. Li still exists stably in oxide films. The APT results also showed that the aggregation of Li at grain boundaries was not affected by injecting boron. Calculations and experimental results suggest that the injection of boric acid did not change the steps 1–4 mentioned above. In addition, B infiltrated into oxide films and migrated to O/M interface, without reducing the aggregation of Li in columnar crystals. The size of B^3+^ is 27 pm, which is much smaller than that of Li^+^, Zr^4+^, and O^2-^. So, B^3+^ can be easily incorporated in the ZrO_2_ lattice, affecting steps 5–6. The corrosion inhibition mechanism of injecting boron is shown in Figure 10b: Li^+^, OH^−^ and B^3+^ diffuse from aqueous solutions to the top of columnar crystal. When Li^+^ replaces Zr^4+^ of the ZrO_2_ lattice, B^3+^ also incorporated in the ZrO_2_ lattice, and its three positive charges balance the loss of positive charges caused by Li^+^ replacing Zr^4+^. It maintains the electrical neutrality of ZrO_2_. Therefore, the generation of oxygen vacancies is suppressed, resulting in insufficiency of oxygen vacancies in oxides, which can transport oxygen to O/M interface, thereby slowing down corrosion. Due to the limited dissociation of boric acid in high-temperature water, it is necessary to inject sufficient boric acid to provide sufficient B^3+^. When the boric acid concentration exceeds a certain degree, the B^3+^ in the ZrO_2_ lattice will reach saturation, and corrosion inhibition effect will reach the upper limit. Therefore, when the B concentration increases from 50 mg/kg to 200 mg/kg, the improvement in the corrosion inhibition effect is much smaller than that when B concentration increases from 0 to 50 mg/kg.

## 4. Conclusions

To clarify the influence of injecting boron on the corrosion resistance of the Zr-Sn-Nb alloy, the 360 °C/18.6 MPa autoclave corrosion test was performed in concentrated LiOH solutions with different boron concentrations. Corrosion resistance and the hydrides’ absorption behavior were obtained. SEM, TEM, GDOES, and APT were used to characterize the oxide films in different solutions. An attempt has been made to explain the accelerated corrosion mechanism induced by the Li mechanism and the corrosion inhibition mechanism of injecting boron.

1. In B = 0 mg/kg solutions, Zr-Sn-Nb alloys were corroded severely, and oxide films showed significant cracking after 180 days. After 510 days, the weight gain was 10879.01 mg/dm^2^. After injecting 50 mg/kg and 200 mg/kg boron in LiOH solutions, the surface of oxide films maintained uniform and dense throughout the entire test. After 510 days, the weight gain increased to 202.38 mg/dm^2^ in B = 50 mg/kg solutions, and 184.77 mg/dm^2^ in B = 200 mg/kg solutions. Injecting boron significantly reduced the corrosion rate, hydrogen concentration, and length of Zr-Sn-Nb alloys.
2. The accelerated corrosion mechanism induced by the Li is as follows: Li^+^ tends to be incorporated in oxides in an alkaline environment, leading to the generation of a large number of oxygen vacancies. Oxygen vacancies carry oxygen from the solutions to the O/M interface, accelerating corrosion.
3. The corrosion inhibition mechanism of injecting boron is as follows: after B^3+^ incorporated in oxides films, the generation of oxygen vacancies is inhibited. This leads to insufficiency of oxygen vacancies, thereby slowing down corrosion. However, when boron concentration exceeds the critical value, the B^3+^ incorporated in the ZrO_2_ lattice will reach saturation, and corrosion inhibition effect will reach the upper limit. Continuing to increase the boron concentration cannot significantly improve corrosion inhibition effect.

The results of this work can do some help to clarify the influence of the boron content in LiOH solution on corrosion resistance of Zr-Sn-Nb alloy, and can provide reference for the optimization of primary coolant in PWRs.

## Figures and Tables

**Table 1:** Range of Zr-Sn-Nb alloy composition (wt%).

Element	Sn	Nb	Fe	Cr	Zr
Content	0.9–1.2	0.25–0.35	0.3–0.4	0.05–0.10	Bal.

**Table 2:** Aqueous solutions compared in this study (mg/kg).

Type of Solutions	Li Concentration	B Concentration	Dissolved Oxygen
B = 0 mg/kg	100 ± 5	0	≤0.1
B = 50 mg/kg	100 ± 5	50 ± 5	≤0.1
B = 200 mg/kg	100 ± 5	200 ± 10	≤0.1

**Table 3:** Hydrogen absorption concentration of Zr-Sn-Nb alloy corroded in different solutions at different times (mg/kg).

	Times	30 Days	90 Days	180 Days	270 Days	360 Days	450 Days	510 Days
Solutions								
B = 0 mg/kg		18	400	2400	3700	3500	3800	3900
B = 50 mg/kg		14	18	20	34	44	58	74
B = 200 mg/kg		20	16	14	28	40	46	49

**Table 4:** The electron spectroscopy results of positions 1, 2, and 3 in Figure 6e (wt%).

Position	O	Cr	Fe	Zr	Nb	Sn
1	2.21	0.00	0.07	96.84	0.41	0.46
2	11.77	0.08	0.06	86.77	0.07	1.26
3	36.83	0.00	0.04	61.94	0.22	0.97

**Table 5:** The electron spectroscopy results of positions 1, 2, and 3 in Figure 7b (wt%).

Position	O	Cr	Fe	Zr	Nb	Sn
1	2.59	0.43	2.10	93.11	1.77	0.00
2	5.78	0.03	0.03	91.34	0.39	2.43
3	23.39	0.00	0.03	75.48	0.19	0.92

**Table 6:** PH_360°C_, undissolved LiOH, Li^+^, and corrosion resistance of Zr-Sn-Nb alloys indifferent solutions.

Serial Number	Li Concentration (mg/kg)	B Concentration (mg/kg)	pH_360°C_	Undissolved LiOH (mol/L)	Li^+^ Concentration (mg/kg)	Corrosion Accelerated?
1	70	0	10.56	4.53 × 10^−7^	≈56	yes
2	70	100	10.44	3.41 × 10^−7^	≈70	no
3	70	1000	9.83	8.30 × 10 ^−8^	≈70	no
4	100	0	10.76	1.13 × 10^−6^	≈100	yes
5	100	50	10.71	1.00 × 10 ^−6^	≈100	no
6	100	200	10.56	7.15 × 10 ^−7^	≈100	no
7	300	0	11.05	4.22 × 10^−6^	≈300	yes
8	300	100	10.98	3.64 × 10^−6^	≈300	no
9	300	1000	10.44	1.05 × 10^−6^	≈300	no
10	700	0	11.30	1.33 × 10^−5^	≈700	yes
11	700	100	11.26	1.23 × 10^−5^	≈700	yes
12	700	200	11.23	1.13 × 10^−5^	≈700	yes
13	700	1000	10.90	5.27 × 10^−6^	≈700	no
14	700	2000	10.51	2.17 × 10^−6^	≈700	no

## Data Availability

The data presented in this study are available on request from the corresponding author due to confidentiality rules.

## References

[1] Duan Z., Yang H., Satoh Y., Murakami K., Kano S., Zhao S., Shen J., Abe H. (2017). Current status of materials development of nuclear fuel cladding tubes for light water reactors. Nucl. Eng. Des..

[2] Wu Z., Jia Y., Dai X., Yi W. (2022). Effect of Reprocess on Microstructure and Corrosion Resistance of Zr-Sn-Nb Alloy. Met..

[3] Motta A., Couet A., Comstock R. (2015). Corrosion of zirconium alloys used for nuclear fuel cladding. Annu. Rev. Mater. Res..

[4] Yu Z., Werden J.W., Capps N.A., Linton K.D., Couet A. (2021). (S)TEM/EDS study of native precipitates and irradiation induced Nb-rich platelets in high-burnup M5. J. Nucl. Mater..

[5] Zhou B., Yao M., Li Z., Wang X., Zhou J., Long C., Liu Q., Luan B. (2012). Optimization of N18 zirconium alloy for fuel cladding of water reactors. J. Mater. Sci. Technol..

[6] Chen Z., Zhao Y., Gong B., Tang M., Wu Z. (2022). Influence of Ammonia on the Corrosion Behavior of a Zr-Sn-Nb Alloy in High Temperature Water. Front. Mater..

[7] Jeong Y., Baek J., Kim S. (1999). Corrosion characteristics and oxide microstructure of Zircaloy-4 in aqueous alkali hydroxide solution. J. Nucl. Mater..

[8] Han J., Rheem K. (1994). The corrosion characteristics of Zircaloy-4 fuel cladding in LiOH-H_3_BO_3_ solutions. J. Nucl. Mater..

[9] Cox B., Wu C. (1995). Transient effects of lithium hydroxide and boric acid on zircaloy corrosion. J. Nucl. Mater..

[10] Park J., Seung J., Choi B., Yong H. (2008). Corrosion and oxide characteristics of Zr-1.5Nb-0.4Sn-0.2Fe-0.1Cr alloys in 360 °C pure water and LiOH solution. J. Nucl. Mater..

[11] Yilmazbayhan A., Breval E., Motta A.T., Comstock R.J. (2006). Transmission electron microscopy examination of oxide layers formed on Zr alloys. J. Nucl. Mater..

[12] Yao M., Zhou B., Li Q., Liu W., Geng X., Lu Y. (2008). A superior corrosion behavior of zircaloy-4 in lithiated water at 360 °C/18.6 mpa by beta-quenching. J. Nucl. Mater..

[13] Oskarsson M., Ahlberg E., Pettersson K. (2001). Oxidation of Zircaloy-2 and Zircaloy-4 in water and lithiated water at 360 °C. J. Nucl. Mater..

[14] Xie S., Zhou B., Liang X., Li Q., Zhang J. (2020). The distribution of Li ions in the oxide film formed on Zircaloy-4 corroded in lithiated water at 633 k. Materials.

[15] Wei K., Wang X., Zhu M., Guan H., Zhang J. (2020). Effects of Li, B and H elements on corrosion property of oxide films on ZIRLO alloy in 300°C/14MPa lithium borate buffer solutions. Corros. Sci..

[16] Kim T., Choi K., Yoo S., Lee Y. (2018). Influence of dissolved hydrogen on the early stage corrosion behavior of zirconium alloys in simulated light water reactor coolant conditions. Corros Sci..

[17] Cox B., Wu C. (1993). Dissolution of zirconium oxide films in 300 °C LiOH. J. Nucl. Mater..

[18] (2011). Standard Test Method for Corrosion Testing of Products of Zirconium, Hafnium, and Their Alloys in Water at 680°F(360°C) or in Steam at 750°F (400°C).

[19] Liu J., Yu H., Karamched P., Hu J., Grovenor C. (2019). Mechanism of the α-Zr to hexagonal-ZrO transformation and its impact on the corrosion performance of nuclear Zr alloys. Acta Mater..

[20] Polatidis E., Frankel P., Wei J., Klaus M., Comstock R., Ambard A., Lyon S., Cottis R., Preuss M. (2013). Residual stresses and tetragonal phase fraction characterisation of corrosion tested zircaloy-4 using energy dispersive synchrotron x-ray diffraction. J. Nucl. Mater..

[21] Pecheur D., Godlewski J., Billot P., Thomazet J. (1996). Microstructure of oxide films formed during the waterside corrosion of the zircaloy-4 cladding in lithiated environment. Zirconium in the Nuclear Industry: 15th International Symposium.

[22] Proff C., Abolhassani S., Lemaignan C. (2013). Oxidation behaviour of zirconium alloys and their precipitates—A mechanistic study. J. Nucl. Mater..

[23] Ni N. (2011). Study of Oxidation Mechanisms of Zirconium Alloys by Electron Microscopy. Ph.D. Thesis.

[24] Igawa N., Ishii Y. (2001). Crystal structure of metastable tetragonal zirconia up to 1473 K. J. Am. Ceram. Soc..

[25] Katz G. (1971). X-ray diffraction powder pattern of metastable cubic ZrO_2_. J. Am. Ceram. Soc..

[26] Cao X., Yao M., Peng J., Zhou B. (2011). Corrosion behavior of Zr(Fe_x_,Cr_1-x_)_2_ alloys in 400 °C superheated steam. Acta Metall. Sin. (Engl. Lett.).

[27] Hu J., Aarholt T., Setiadinata B., Li K., Grovenor C. (2019). A multi-technique study of “barrier layer” nano-porosity in Zr oxides during corrosion and hydrogen pickup using (S)TEM, TKD, APT and Nano SIMS. Corros. Sci..

[28] Xie S., Zhou B., Liang X., Liu W., Li H., Li Q., Yao M., Zhang J. (2017). A novel mechanism for nodular corrosion of zircaloy-4 corroded in 773 K superheated steam. Corros. Sci..

[29] Macdonald D. (2012). Some personal adventures in passivity—A review of the point defect model for film growth. Russ. J. Electrochem..

[30] Hu J., Liu J., Lozano-Perez S., Grovenor C., Mader E. (2019). Hydrogen pickup during oxidation in aqueous environments: The role of nano-pores and nano-pipes in zirconium oxide films. Acta Mater..

[31] Wei K., Chen L., Qu Y., Zhang Y., Zhang J. (2018). Zeta potential of microarc oxidation film on ZIRLO alloy in different aqueous solutions. Corros. Sci..

[32] Krausová A., Macák J., Sajdl P., Novotny R., Reniuková V., Vrtílková V. (2015). In-situ electrochemical study of Zr-1Nb alloy corrosion in high temperature Li+ containing water. J. Nucl. Mater..

[33] Liu W., Zhou B., Li Q., Yao M. (2005). Detrimental role of LiOH on the oxide film formed on Zircaloy-4. Corros. Sci..

